# DGRO: Doppler Velocity and Gyroscope-Aided Radar Odometry

**DOI:** 10.3390/s24206559

**Published:** 2024-10-11

**Authors:** Chao Guo, Bangguo Wei, Bin Lan, Lunfei Liang, Houde Liu

**Affiliations:** 1 Jianghuai Advanced Technology Center, Hefei 230031, China; 2 Harbin Institute of Technology, Harbin 150001, China; 3 Tsinghua Shenzhen International Graduate School, Shenzhen 518055, China

**Keywords:** 4D mm wave radar, radar odometry, ego-velocity estimation, gyroscope

## Abstract

A stable and robust odometry system is essential for autonomous robot navigation. The 4D millimeter-wave radar, known for its resilience in harsh weather conditions, has attracted considerable attention. As the latest generation of FMCW radar, 4D millimeter-wave radar provides point clouds with both position and Doppler velocity information. However, the increased uncertainty and noise in 4D radar point clouds pose challenges that prevent the direct application of LiDAR-based SLAM algorithms. To address this, we propose a SLAM framework that fuses 4D radar data with gyroscope readings using graph optimization techniques. Initially, Doppler velocity is employed to estimate the radar’s ego velocity, with dynamic points being removed accordingly. Building on this, we introduce a pre-integration factor that combines ego-velocity and gyroscope data. Additionally, leveraging the stable RCS characteristics of radar, we design a corresponding point selection method based on normal direction and propose a scan-to-submap point cloud registration technique weighted by RCS intensity. Finally, we validate the reliability and localization accuracy of our framework using both our own dataset and the NTU dataset. Experimental results show that the proposed DGRO system outperforms traditional 4D radar odometry methods, especially in environments with slow speeds and fewer dynamic objects.

## 1. Introduction

SLAM is a core technology in autonomous driving and robotics. Currently, most platforms use LiDAR or cameras as their primary sensors and related SLAM algorithms have demonstrated high performance [1,2,3,4,5]. However, in adverse weather conditions such as fog, haze, rain or snow, the positioning performance of these sensors can degrade significantly or even fail. In contrast, millimeter-wave radar can maintain stable and reliable performance under various harsh conditions due to its insensitivity to weather, lighting, and smoke [6]. Although millimeter-wave radar is more cost-effective compared to LiDAR, its application in SLAM is still underdeveloped [7].

The 4D millimeter-wave radar (4D radar) enhances ordinary 3D millimeter-wave radar (3D radar) by adding the capability to measure elevation angles, allowing it to output target distance, azimuth, Doppler velocity, and RCS information, with a higher point cloud density than 3D radar. However, most 3D radar positioning algorithms rely on mapping planar points into images and using image-based methods, which are not suitable for 4D radar [8,9]. Additionally, compared to LiDAR, the point clouds generated by 4D radar are sparser and noisier, making efficient positioning through direct feature matching challenging. Furthermore, issues such as multipath effects, harmonics, and inherent sensor limitations introduce significant noise into the scanned point clouds [10]. Consequently, developing a robust and high-performance SLAM algorithm for 4D radar remains a significant challenge.

We propose a novel SLAM approach that integrates radar scan point clouds, Doppler, and gyroscope data. The proposed method, DGRO, achieves accurate odometry and consistent map construction by fusing Doppler velocities from radar scans, scan-to-submap matching, and gyroscope data. Our contributions include the following:

1. Ego-Velocity Preintegration: By leveraging the radar’s velocity derived from Doppler measurements and directional information from the gyroscope, along with a pre-integration method similar to IMU pre-integration, we obtain a robust estimate of the pose and odometry, which provides a reliable initial estimate for scan-to-submap matching.
2. Scan-to-Submap Registration: Before registration, we filter the points using RCS and normal vectors to select more accurate correspondences, and then apply an RCS-weighted ICP algorithm for registration. The factor graph integrates Doppler-IMU pre-integration factors, scan-to-submap registration factors, and loop closure factors, enabling precise positioning even in the absence of scan point clouds for short periods.
3. Experimental Validation: Extensive experiments on our platform using five datasets demonstrate the accuracy, robustness, and real-time performance of the proposed method.

The rest of the paper is organized as follows: Section 2 reviews the related work, and Section 3 describes the methodology, including the design of the DGRO system. Section 3 covers ego-velocity estimation (Section 3.1), ego-velocity pre-integration (Section 3.2), scan-to-submap matching (Section 3.3), and loop closure detection (Section 3.4). Section 4 presents the experimental setup and results, and Section 5 concludes the paper with final remarks and potential future work.

## 2. Related Work

This section provides a concise overview of recent work on 4D radar odometry and 4D radar-IMU fusion odometry. Compared to LiDAR, 4D radar generates sparser and noisier point clouds, leading to adaptations in 4D radar odometry algorithms. These adaptations often involve modifications to the feature extraction or point cloud registration processes, initially developed for LiDAR SLAM, to better handle the unique characteristics of radar data.

### 2.1. 4D Radar Odometry

One of the key advantages of radar is its ability to measure the Doppler velocity of each target point, which can enhance the performance of radar odometry. Zhang et al. [11] introduced a 4D Radar SLAM approach that utilizes APDGICP for point cloud registration. They modified the GICP algorithm [12] to better suit radar point clouds and estimated the radar’s velocity using Doer’s method [13], providing an initial estimate for APDGICP. This method performs well in low-speed outdoor scenarios but struggles at higher speeds. Wu et al. [14] proposed EFEAR-4D, which clusters Doppler velocities using the DBSCAN algorithm [15] to estimate the radar’s velocity, providing a reliable initial estimate for point cloud matching. By removing dynamic objects and extracting robust regional features, EFEAR-4D maintains strong performance across various environments with sparse and noisy point clouds. However, its effectiveness decreases significantly during rapid rotational movements. Li et al. [16] proposed a 4D Radar SLAM framework that optimizes pose graphs. To mitigate the effects of multipath interference and noise, they filtered the raw 4D radar data and designed a pre-integration factor based on the radar’s linear and angular velocities, resulting in more accurate and robust pose estimates. However, this method requires careful consideration of the radar’s installation position, as improper placement can hinder the accurate estimation of angular velocity.

### 2.2. 4D Radar-IMU Odometry

IMUs provide high-frequency motion data, which, despite accumulating significant errors over time, offer highly accurate short-term relative displacement measurements. Thus, when other sensors fail, integrating IMU data can significantly enhance positioning accuracy and improve the overall performance and robustness of the SLAM system [17]. Doer et al. [13] developed a radar inertial odometry (RIO) system based on an Extended Kalman Filter (EKF) [18], using Doppler velocity measurements from the radar. They employed the RANSAC algorithm [19] to estimate the radar’s velocity and integrated inertial data to achieve precise and robust odometry. Michalczyk et al. [20] proposed a tightly coupled RIO approach that compares the distances of matched feature points with current radar measurements and uses radar-derived velocity information to correct IMU drift. Their method significantly outperforms traditional techniques, particularly in managing noisy and sparse radar point cloud data. Zhuang et al. [21] utilized EKF to integrate scan-to-submap registration constraints and radar velocity estimates for odometry and mapping. They employed ScanContext [22] for loop closure detection and achieved efficient SLAM through GICP [12] and pose graph optimization. While 4D radar point clouds have become denser, enabling position constraints between successive point cloud frames through matching, current methods remain sensitive to noise and the uncertainty of scanned points. This sensitivity can lead to failures in areas with sparse point clouds, potentially causing system crashes [23]. Doppler measurements, which directly assess velocity, can accurately estimate displacement even in repetitive environments, unaffected by external conditions. Moreover, compared to accelerometers, displacement estimation through velocity integration exhibits lower cumulative errors and drift [24]. Based on these insights, we propose a SLAM system that integrates Doppler velocity, gyroscope data, and point cloud registration.

## 3. Methodology

**Figure 1:**
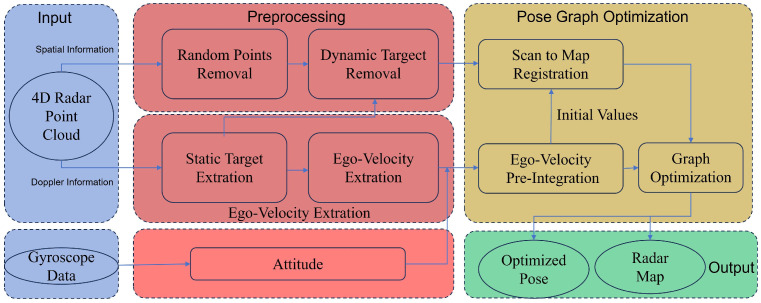
Overview of the proposed DGRO system.

Figure 1 presents an overview of the DGRO system, which processes inputs from both 6D IMU data and radar scan point clouds. Initially, the point cloud data undergo preprocessing to filter out dynamic objects and noise. Following this, the radar’s velocity is estimated using Doppler data, which is then combined with the IMU’s attitude information. A relative pose transformation is subsequently calculated using a method akin to pre-integration, providing a robust initial estimate for the scan-to-submap matching process. This matching leverages constraints based on point normals, distances, and Radar Cross Section (RCS) values to enhance both speed and accuracy. Loop closure detection on keyframes is handled by Quatro++ [25]. Finally, gtsam [26] is employed to construct a factor graph and perform optimization, resulting in a globally consistent map and optimized pose estimates.

### 3.1. Ego-Velocity Estimation

4D Radar measurements offer comprehensive data, including both the spatial positions of points and their Doppler velocities. Doppler velocity indicates the radial speed of a target relative to the radar.

In a typical data frame, most of the points are stationary. Given the position of a target point n as $p^{r}$, the normalized direction of the target is defined as:

(1) $$r^{r}=\frac{p^{r}}{∥p^{r}∥},$$

In a global coordinate system, the Doppler velocity $v_{d}^{r}$ of a static point is opposite in direction to the radar’s velocity. Thus, the radar’s velocity $v_{d}^{r}$ can be determined by taking the negative product of the static point’s Doppler velocity $v^{r}$ and the direction vector $r^{r}$:

(2) $$v_{d}^{r}=-{\left(v^{r}\right)}^{⊤}r^{r},$$

Given the Doppler velocities of *n* points, the radar’s velocity can be estimated using the least squares method:

(3) $$v^{r}=\arg\min_{v^{r}}\sum_{i=1}^{n}{∥v_{d}^{r}+{\left(v^{r}\right)}^{⊤}r_{i}^{r}∥}^{2},$$

Since dynamic points are inevitably present among these *n* points, directly applying the least squares method might produce inaccurate results. To address this, RANSAC is used to filter out outliers and provide a more reliable estimation of the radar’s velocity $v^{r}$. This approach not only improves the accuracy of the radar velocity estimation but also helps in distinguishing dynamic targets from static ones. Consequently, during subsequent point cloud registration, only static points are used for matching, leading to enhanced registration performance.

### 3.2. Ego-Velocity Preintegration

Radar velocity is crucial for motion constraints. Inspired by IMU pre-integration [27], we fuse the Doppler velocity measured by the radar at time *i* with the attitude information from the gyroscope to estimate the radar’s relative pose at time *j*. For simplicity, we assume that the IMU coordinate system aligns with the vehicle coordinate system.

At time *t*, the estimated velocity, including the gyroscope’s angular velocity ${\tilde{\omega }}_{t}$ and the radar’s linear velocity ${\tilde{v}}_{t}^{i}$ in the gyroscope coordinate system, is subject to time-varying white noise $\eta_{k}^{w}$ and $\eta_{k}^{v}$.

(4) $${\tilde{\omega }}_{t}=\omega_{t}+\eta_{t}^{\omega },$$

(5) $${\tilde{v}}_{t}^{b}=R_{r}^{b}v_{t}^{r}+\eta_{t}^{v},$$

Radar velocity is pre-integrated between adjacent keyframes. During the interval between discrete times *i* and *j*, there are *n* IMU frames with a time interval of Δt between adjacent frames, and *m* radar frames with a time interval of aΔt between adjacent frames. Each radar frame’s velocity is denoted as ${\tilde{v}}_{q}^{i}$, where q∈[1,m]. It is assumed that the radar moves at a constant velocity between successive radar frames. Given that the gyroscope frequency is typically much higher than the radar frequency, there are several gyroscope frames between each pair of radar frames. Here, *a* represents the number of gyroscope frames within a single radar frame’s time interval Δt.

(6) $$R_{\left(t+a\Delta t\right)}=R_{t}\prod_{k=t}^{t+a\Delta t}\mathrm{Exp}\left(\omega_{k}\Delta t\right),$$

(7) $$p_{\left(t+a\Delta t\right)}=p_{t}+{\tilde{v}}_{q}^{i}a\Delta t,$$

By substituting Equations (4) and (5) into Equations (6) and (7), respectively, the following results are obtained:

(8) $$R_{\left(t+a\Delta t\right)}=R_{t}\prod_{k=t}^{t+a\Delta t}\mathrm{Exp}\left(({\tilde{\omega }}_{k}-\eta_{t}^{\omega })\Delta t\right),$$

(9) $$p_{\left(t+a\Delta t\right)}=p_{t}+\left({\tilde{v}}_{t}^{b}-\eta_{t}^{v}\right)\Delta t,$$

Thus, the direction $R_{j}$ and position $p_{j}$ of the vehicle at time *j* can be determined as follows:

(10) $$R_{j}=R_{i}\prod_{k=i}^{j-1}\mathrm{Exp}\left(({\tilde{\omega }}_{k}-\eta_{k}^{\omega })\Delta t\right),$$

(11) $$p_{j}=p_{i}+\sum_{k=i}^{j-1}R_{k}\left({\tilde{v}}_{t}^{b}-\eta_{k}^{v}\right)\Delta t,$$

Using the equation above, the relative rotation between keyframes and can be computed as follows:

(12) $$\Delta R_{ij}=R_{i}^{⊤}R_{j}=\prod_{k=i}^{j-1}\mathrm{Exp}\left(({\tilde{\omega }}_{k}^{b}-\eta_{k}^{\omega })\Delta t\right)=\underset{{\tilde{\Delta R}}_{ij}}{\underset{︸}{\prod_{k=i}^{j-1}\mathrm{Exp}\left({\tilde{\omega }}_{k}\Delta t\right)}}\underset{\mathrm{Exp}(-\delta \phi_{ij})}{\underset{︸}{\prod_{k=i}^{j-1}\mathrm{Exp}\left(-{\tilde{\Delta R}}_{\left(k+1,j\right)}^{⊤}J_{r,k}\eta_{k}^{\omega }\Delta t\right)}},$$

The relative translation between keyframes can be determined as follows:

(13) $$\Delta p_{ij}=R_{i}^{⊤}\left(p_{j}-p_{i}\right)=\sum_{k=i}^{j-1}\Delta R_{ik}\left({\tilde{v}}_{t}^{b}-\eta_{k}^{v}\right)\Delta t=\underset{{\tilde{\Delta P}}_{ij}}{\underset{︸}{\sum_{k=i}^{j-1}{\tilde{\Delta R}}_{ik}{\tilde{v}}_{t}^{b}\Delta t}}-\underset{\delta p_{ij}}{\underset{︸}{\sum_{k=i}^{j-1}\left(-{\tilde{\Delta R}}_{ik}{\left(v_{k}^{b}\right)}^{\wedge }\delta \phi_{ik}\Delta t+{\tilde{\Delta R}}_{ik}\eta_{k}^{v}\Delta t\right)}},$$

Here, $\Delta R_{ik}=R_{i}^{⊤}R_{k}$, and $J_{r,k}$ represents the right Jacobian matrix of ${\tilde{\omega }}_{k}^{b}$ (for detailed derivation, refer to [17]). The matrix ${\tilde{v}}_{k}^{b^{\wedge }}$ is the skew-symmetric matrix of the vector ${\tilde{v}}_{k}^{b}$. The errors $\Delta R_{ij}$ and $\Delta p_{ij}$ in $\delta \phi_{ij}$ and $\delta p_{ij}$ are linear transformations, so their errors, like those in $\eta_{t}^{\omega }$ and $\eta_{t}^{v}$, follow a zero-mean Gaussian distribution. Since the frequency of ${\tilde{\omega }}_{t}$ during the interval [i,j] is significantly higher than that of ${\tilde{v}}_{t}^{b}$, the most recent rotation increment ${\tilde{\Delta R}}_{ik}$ is used in the displacement pre-integration calculations.

The residual of the pre-integrated velocity can be expressed as:

(14) $$r_{\Delta R_{ij}}=\log\left({\tilde{\Delta R}}_{ij}^{⊤}\left(R_{i}^{⊤}R_{j}\right)\right),$$

(15) $$r_{\Delta p_{ij}}=R_{i}^{⊤}\left(p_{j}-p_{i}-v_{i}^{b}\Delta t_{ij}-\frac{1}{2}g\Delta t_{ij}^{2}\right)-{\tilde{\Delta p}}_{ij},$$

In this context, Log(·) denotes the logarithmic map from SO(3) to $R^{3}$.

### 3.3. Scan to Sub-Map Matching

The radar cross section (RCS) of millimeter-wave radar, which is influenced by the material, shape, and surface roughness of the target, holds significant importance in measurement results. Given the sparsity of millimeter-wave radar point clouds, direct inter-frame matching can lead to substantial errors or even registration failure. To address this, we employ a submap matching approach between the current frame and key frames. By utilizing an enhanced ICP method, accurate pose transformation matrices are derived through precise point cloud registration. The detailed methodology is as follows:

The transformation matrix between frames, obtained through Ego-Velocity Preintegration, serves as the initial estimate for point cloud registration. Here, $P^{r}$ represents the point cloud of the current frame, while $Q^{r}$ denotes the submap point cloud, with points $p_{i}\in P^{r}$ belonging to $q_{j}\in Q^{r}$, where i,j=1,2,…. Before proceeding with the registration, valid point pairs are filtered based on distance and RCS thresholds to eliminate incorrect matches. Using a Kd-tree, the nearest point $q_{k1}$ to each point $p_{i}$ in the submap point cloud is identified. Subsequently, the cosine of the angle $\cos\theta_{i}$ between the normal vector $n_{i}$ of $q_{i}$ and the normal vector $n_{ki}$ of $p_{i}$ is calculated. The local surface normal vector is estimated by calculating the covariance matrix on the neighborhood point set and performing eigenvalue decomposition, with the eigenvector corresponding to the smallest eigenvalue being taken as the normal vector [28].

(16) $$\cos\theta_{i}=\frac{n_{i}\cdot n_{ki}}{∥n_{i}∥∥n_{ki}∥},$$

The Euclidean distance between the points $p_{i}$ and $q_{i}$ is given by the following:

(17) $$d_{i}={∥q_{i}-p_{i}∥}_{2},$$

The difference in RCS between the points $p_{i}$ and $q_{i}$ is calculated as follows:

(18) $$d_{i}^{\mathrm{power}}=\left|q_{i}^{\mathrm{power}}-p_{i}^{\mathrm{power}}\right|,$$

The point pair $\left(p_{i},q_{k1}\right)$ is considered valid if it simultaneously satisfies the following conditions:

(19) $$\left\{\begin{array}{c} \cos\theta_{i}\geq c\\ d_{i}\leq d\\ d_{i}^{\mathrm{power}}\leq d^{\mathrm{power}} \end{array}\right.,$$

where *c*, *d*, and $d^{\mathrm{power}}$ are constants. Based on our tests, setting the threshold to one-quarter of the maximum RCS achieves a good balance between the accuracy and speed of point cloud registration. If the threshold is too high, the number of iterations in the ICP algorithm tends to increase, leading to decreased computational efficiency; conversely, if the threshold is too low, the ICP may fail to converge.

Once the valid points are determined, a weighted least squares function is formulated as follows:

(20) $$\underset{T_{k}^{s}}{\mathrm{argmin}}\sum_{i=1}^{n}\omega_{i}{∥p_{i}-T_{k}^{s}q_{i}∥}^{2},$$

where

(21) $$\omega_{i}=\frac{d_{i}^{\mathrm{power}}-d_{\min}^{\mathrm{power}}}{d_{\max}^{\mathrm{power}}-d_{\min}^{\mathrm{power}}},$$

*n* denotes the number of corresponding point pairs, while $d_{\min}^{\mathrm{power}}$ and $d_{\max}^{\mathrm{power}}$ represent the minimum and maximum RCS values, respectively.

**Figure 2:**
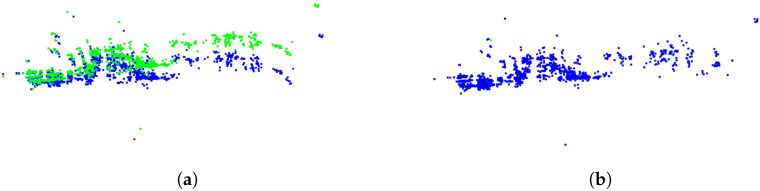
Results of registering two radar point clouds using RCS-ICP. (**a**) Initial point clouds, (**b**) Results after registration.

In Figure 2, (a) shows the two radar point clouds in green and blue, representing different frames. (b) displays the result after registration, where the two point clouds almost completely overlap.

Furthermore, when the positional or rotational differences between consecutive point clouds surpass a specified threshold, the corresponding frame is identified as a keyframe and subsequently added to the keyframe set.

### 3.4. Loop Closure Detection

The integration of loop closure detection significantly improves the robustness of the entire SLAM system and enhances the accuracy of localization. Upon detecting each new radar keyframe, Quatro++ is employed to extract FPFH features from both the historical keyframes and the new keyframe, followed by coarse matching [29]. The historical keyframe that exhibits the minimal registration error is identified as a valid loop closure. The result of this coarse registration serves as the initial guess for the registration method discussed in Section 3.3. A more precise registration is then performed, and the resulting relative pose is incorporated as a loop closure factor into the factor graph, thereby refining the overall graph optimization results.

## 4. Experiments

### 4.1. Quantitative Evaluation

**Figure 3:**
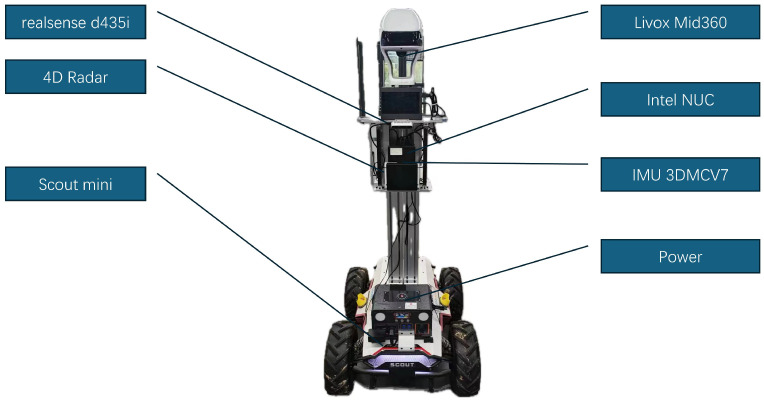
The ground robot and the sensors for tests. Note that the realsense camera and the RGBD camera are not used.

The mobile robot was equipped with the Altos V1 4D radar, which operates at a scanning frequency of 10Hz and within a frequency range of 76–81 GHz. The radar has a horizontal field of view (HFOV) of ±50° and a vertical field of view (VFOV) of ±11.5°. The azimuth angle resolution and accuracy are 1.32° and 0.14°, respectively, while the elevation angle resolution and accuracy are 1.43° and 0.32°, respectively. The radar can detect objects up to a range of approximately 350 m, with a distance accuracy of 0.1 m. The radar also supports a maximum speed range of −110 m/s to +55 m/s, with a speed resolution of 0.2 m/s and an accuracy of 0.02 m/s. For motion sensing, we utilized the Lord 3DMCV7 IMU, which provides precise pose estimation through its gyroscope. The IMU data are fused with radar measurements to enhance the overall system’s accuracy. The gyroscope features an angular random walk of $0.14^{\circ }/\sqrt{h}$ and a bias instability of 1.5°/h. All our experiments were conducted offline but at real-time speed, processing the recorded sensor data on a custom C++ framework running on an Intel Core i7-1360P NUC computer with 32 GB RAM. The layout of the robot and the positioning of the sensors are depicted in the accompanying Figure 3.

**Figure 4:**
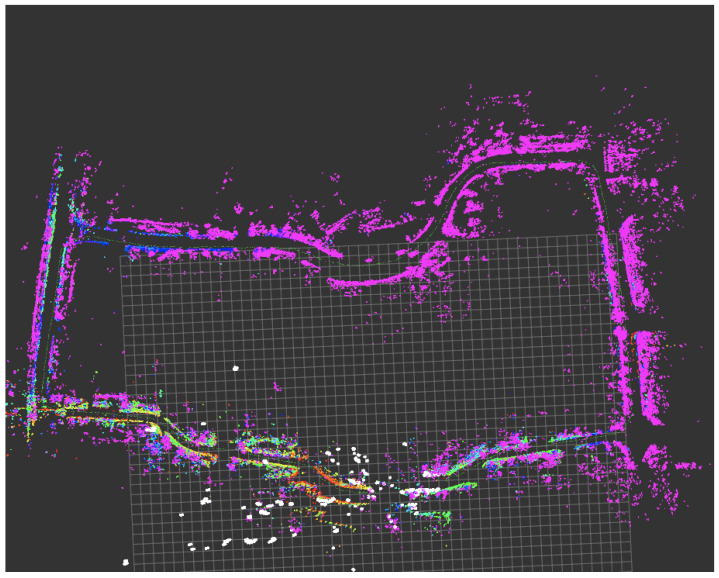
Mapping results by DGRO on data of a large-scale industrial park.

We collected data in two outdoor scenarios in Hefei, at Zhong’an Chuanggu Phase II. Sequence 1 involves a loop around Building H6, where the GPS signal is strong, with a collection speed of approximately 3 m/s. The starting and ending poses are the same. Sequence 2 involves a loop around the lawn square, where high-rise buildings on both sides occasionally cause GPS signal interruptions, with a collection speed of approximately 2 m/s. The starting and ending poses are the same. For Sequence 1, we used centimeter-level RTK (Real-Time Kinematic) data as the ground truth for trajectory evaluation. In Sequence 2, we used odometry data obtained from Fast-LIO2 [30], which integrates LiDAR and IMU data, as the reference. All sensors involved in the experiments were pre-calibrated for their extrinsic parameters to ensure accuracy. To assess the performance of our algorithm, we employed the open-source tool EVO Trajectory Evaluation [31] to compute the Absolute Trajectory Error (ATE) and Relative Error (RE). The ATE measures the absolute difference between the estimated and true trajectories, while the RE quantifies the consistency of trajectory estimates relative to each other. To facilitate evaluation, we compared the relative error (RE) and absolute trajectory error (ATE) of Our Odometry (without loop closure) and Our SLAM against the ground truth, as shown in Table 1. As the table indicates, in Sequence 1, Our Odometry’s relative translation error is 2.40%, relative rotation error is 0.0112 deg/m, and ATE is 4.30 m. Compared to Fast-lio2, both RE and ATE are slightly worse, primarily due to the lower accuracy of the 4D radar point clouds we utilized. Our SLAM system, which incorporates loop closure, achieved a relative translation error of 2.21%, a relative rotation error of 0.0110 deg/m, and an ATE of 2.23 m. Compared to Our Odometry, both RE and ATE improved significantly, approaching the performance of Fast-LIO2. Figure 4 shows the mapping results for Sequence 2.

### 4.2. Ablation Study

In this ablation study, we evaluated our method in comparison to 4DRadarSLAM [11] and using the NTU dataset. The dataset features several scenarios: “garden”, “cp”, and “nyl”, which were collected with a handcar. These scenarios involve relatively low speeds and few dynamic objects, presenting minimal challenges for SLAM algorithms. Due to the lack of ground truth in this dataset, we used odometry results derived from Lidar and IMU data processed with Fast-lio as our reference.

Conversely, the “loop1” and “loop2” scenarios were collected with an in-vehicle platform traveling at speeds up to 30 km/h, encountering significant traffic, thus introducing more substantial challenges for the SLAM systems. For these Sequences, ground truth was obtained using RTK. This study allows us to assess the performance of various SLAM methods under different conditions, from less challenging environments to more complex scenarios involving higher speeds and dense traffic.

In the experiment, we tested apdgicp, apdgicp-lc, RCS-icp, RCS-icp + EVP, and our SLAM system. Here, “lc” stands for loop closure, and “EVP” stands for Ego-Velocity Preintegration. The experimental data are shown in Table 2. (1) When only using point cloud constraints, RCS-icp performs better than Apdgicp in the cp, garden, and ny1 sequences, primarily due to the effectiveness of RCS constraints during matching. However, in the loop1 and loop2 sequences, RCS-icp does not perform as well as apdgicp. This is mainly because the loop sequences were collected on highways, where there are many dynamic vehicles. The high reflectivity of the moving vehicles caused poor matching results, leading to less accurate localization. (2) RCS-icp + EVP showed a significant improvement in accuracy across all five sequences compared to RCS-icp, approaching the accuracy of apdgicp-lc. This indicates that Ego-Velocity Preintegration played a crucial role in the algorithm’s performance. (3) Our SLAM system further improved the accuracy based on RCS-icp + EVP, surpassing the performance of 4DRadarSLAM.

### 4.3. Registration Efficiency

**Figure 5:**
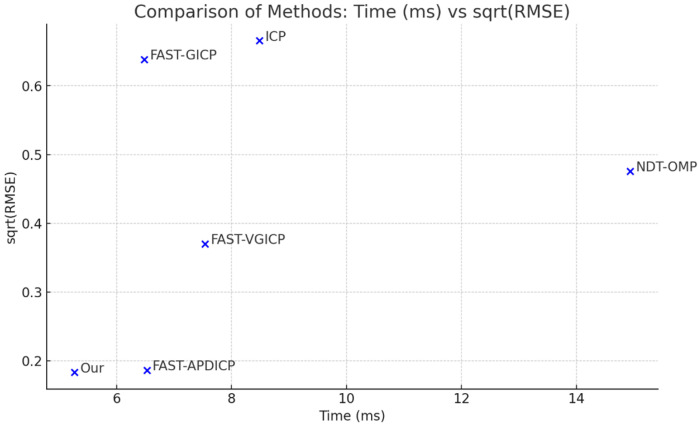
Comparison of accuracy and computational time for different registration algorithms.

In this experiment, we matched the same two frames of point clouds to compare the performance of several registration algorithms. Each algorithm was tested 10 times, and the average result was taken as the final outcome. The algorithms compared include ICP [32], NDT-OMP [33], FAST-GICP [34], FAST-VGICP [34], FAST-APDICP [11], as well as our custom-developed algorithm. The comparison of computation time and accuracy, as shown in Figure 5, reveals that our algorithm significantly reduces processing time while maintaining exceptionally high accuracy compared to other methods. Specifically, with a processing time of only 5.265 milliseconds, our algorithm achieves the lowest RMSE, demonstrating outstanding matching accuracy. Although other algorithms, such as NDT-OMP and FAST-VGICP, also perform well in terms of accuracy, they require longer running times and higher computational resources. In contrast, our algorithm maintains high accuracy while substantially reducing computation time, showcasing its potential for practical applications.

Across all 10 experiments, our algorithm consistently delivered high accuracy and fast matching speed, highlighting its stability and reliability in various scenarios. This positions our algorithm as particularly advantageous for real-time applications. Overall, our algorithm demonstrates significant advantages in both accuracy and efficiency, making it especially suitable for scenarios with strict demands on computational resources and real-time performance.

## 5. Conclusions

This paper presents a novel 4D radar odometry framework that integrates several advanced techniques. We enhance radar self-velocity estimation by employing the RANSAC algorithm to filter outliers from Doppler velocities, leading to accurate velocity estimation. We introduce a Doppler-gyr preintegration method inspired by IMU preintegration principles. Moreover, we enhance the traditional ICP algorithm by incorporating radar RCS information and point cloud normals and apply Quatro++ for robust loop detection.

Our experiments, conducted on both the NTU dataset and a newly collected dataset, demonstrate that our method surpasses current radar odometry techniques in various challenging environments, although it is slightly less effective compared to advanced lidar odometry methods. This positions 4D radar as a promising option for reliable and accurate localization. While our results indicate some limitations in perception under certain conditions, we anticipate these issues can be mitigated by integrating additional complementary sensors. Future work will focus on exploring the uncertainty modeling of radar point clouds and the fusion of 4D radar with other sensors to develop a more comprehensive and effective odometry solution.

## Figures and Tables

**Table 1:** Quantitative analysis of trajectory errors: relative trajectory error ($t_{\mathrm{rel}}$), relative rotation error ($r_{\mathrm{rel}}$), and absolute trajectory error ($t_{\mathrm{abs}}$).

Sequence		Fast-lio2 [30]				Our Odometry				Our SLAM		
		$t_{\mathrm{rel}}$	$r_{\mathrm{rel}}$	$t_{\mathrm{abs}}$		$t_{\mathrm{rel}}$	$r_{\mathrm{rel}}$	$t_{\mathrm{abs}}$		$t_{\mathrm{rel}}$	$r_{\mathrm{rel}}$	$t_{\mathrm{abs}}$
		**(%)**	**(deg/m)**	**(m)**		**(%)**	**(deg/m)**	**(m)**		**(%)**	**(deg/m)**	**(m)**
1		1.83	0.0095	2.15		2.40	0.0112	4.30		2.21	0.0110	2.23
2		-	-	-		2.13	0.0102	3.29		1.92	0.0102	1.93

**Table 2:** Ablation Study of trajectory errors: relative trajectory error ($t_{\mathrm{rel}}$), relative rotation error ($r_{\mathrm{rel}}$), and absolute trajectory error ($t_{\mathrm{abs}}$).

Sequence		apdgicp [11]				apdgicp-lc [11]				RCS-icp				RCS-icp + EVP				Our SLAM		
		$t_{\mathrm{rel}}$	$r_{\mathrm{rel}}$	$t_{\mathrm{abs}}$		$t_{\mathrm{rel}}$	$r_{\mathrm{rel}}$	$t_{\mathrm{abs}}$		$t_{\mathrm{rel}}$	$r_{\mathrm{rel}}$	$t_{\mathrm{abs}}$		$t_{\mathrm{rel}}$	$r_{\mathrm{rel}}$	$t_{\mathrm{abs}}$		$t_{\mathrm{rel}}$	$r_{\mathrm{rel}}$	$t_{\mathrm{abs}}$
		**(%)**	**(deg/m)**	**(m)**		**(%)**	**(deg/m)**	**(m)**		**(%)**	**(deg/m)**	**(m)**		**(%)**	**(deg/m)**	**(m)**		**(%)**	**(deg/m)**	**(m)**
cp		3.57	0.0365	2.66		3.05	0.0442	2.56		3.77	0.0382	2.73		3.02	0.0401	2.49		2.91	0.0398	2.27	
garden		2.38	0.0350	4.02		2.07	0.0390	2.38		2.32	0.0336	3.89		2.05	0.0321	3.82		2.00	0.0301	2.27	
loop1		6.09	0.082	227.54		5.79	0.0131	84.88		7.58	0.088	251.13		3.21	0.0088	55.23		3.05	0.0073	33.2	
Loop2		4.09	0.0097	59.12		4.03	0.0069	43.67		4.12	0.0102	65.38		4.01	0.0062	38.22		3.87	0.0058	26.58	
ny1		3.52	0.0176	22.35		2.83	0.0128	12.35		3.48	0.0168	21.28		2.75	0.0130	13.48		2.30	0.098	8.32	

## Data Availability

Data will be made available on request by email.
